# Genotypic and Phenotypic Characterization of Antimicrobial Resistance in Neisseria gonorrhoeae: a Cross-Sectional Study of Isolates Recovered from Routine Urine Cultures in a High-Incidence Setting

**DOI:** 10.1128/mSphere.00373-19

**Published:** 2019-07-24

**Authors:** Adam L. Bailey, Robert F. Potter, Meghan A. Wallace, Caitlin Johnson, Gautam Dantas, Carey-Ann D. Burnham

**Affiliations:** a Department of Pathology and Immunology, Washington University School of Medicine, St. Louis, Missouri, USA; b The Edison Family Center for Genome Sciences and Systems Biology, Washington University School of Medicine, St. Louis, Missouri, USA; c Department of Molecular Microbiology, Washington University School of Medicine, St. Louis, Missouri, USA; d Department of Biomedical Engineering, Washington University in St. Louis, St. Louis, Missouri, USA; e Department of Medicine, Washington University School of Medicine, St. Louis, Missouri, USA; f Department of Pediatrics, Washington University School of Medicine, St. Louis, Missouri, USA; U.S. Centers for Disease Control and Prevention

**Keywords:** *Neisseria gonorrhoeae*, gonorrhea, urine culture

## Abstract

The objectives of this study were to perform genomic and phenotypic characterization of antimicrobial resistance in Neisseria gonorrhoeae isolates recovered from urine samples from patients in St. Louis, MO, USA. Sixty-four clinical isolates were banked over a 2-year period and subjected to antimicrobial susceptibility testing (AST) by Kirby-Bauer disk diffusion (penicillin, tetracycline, cefuroxime, and ciprofloxacin) and gradient diffusion (tetracycline, doxycycline, azithromycin, ceftriaxone, cefixime, ciprofloxacin, gemifloxacin, and delafloxacin). The medical records for the patients were evaluated to determine the demographics, location, and prescribed treatment regimen. Isolate draft genomes were assembled from Illumina shotgun sequencing data, and resistance determinants were identified by ResFinder and PointFinder. Of the 64 isolates, 97% were nonsusceptible to penicillin, with resistant isolates all containing the *bla*_TEM-1b_ gene; 78 and 81% of isolates were nonsusceptible to tetracycline and doxycycline, respectively, with resistant isolates all containing the *tet*(M) gene. One isolate was classified as non-wild-type to azithromycin, and all isolates were susceptible to ceftriaxone; 89% of patients received this combination of drugs as first-line therapy. Six percent of isolates were resistant to ciprofloxacin, with most resistant isolates containing multiple *gyrA* and *parC* mutations. Correlation between disk and gradient diffusion AST devices was high for tetracycline and ciprofloxacin (*R*^2^ > 99% for both). The rates of N. gonorrhoeae antibiotic resistance in St. Louis are comparable to current rates reported nationally, except ciprofloxacin resistance was less common in our cohort. Strong associations between specific genetic markers and phenotypic susceptibility testing hold promise for the utility of genotype-based diagnostic assays to guide directed antibiotic therapy.

**IMPORTANCE** Neisseria gonorrhoeae causes the sexually transmitted infection gonorrhea, which is most commonly diagnosed using a DNA-based detection method that does not require growth and isolation of N. gonorrhoeae in the laboratory. This is problematic because the rates of antibiotic resistance in N. gonorrhoeae are increasing, but without isolating the organism in the clinical laboratory, antibiotic susceptibility testing cannot be performed on strains recovered from clinical specimens. We observed an increase in the frequency of urine cultures growing N. gonorrhoeae after we implemented a total laboratory automation system for culture in our clinical laboratory. Here, we report on the rates of resistance to multiple historically used, first-line, and potential future-use antibiotics for 64 N. gonorrhoeae isolates. We found that the rates of antibiotic resistance in our isolates were comparable to national rates. Additionally, resistance to specific antibiotics correlated closely with the presence of genetic resistance genes, suggesting that DNA-based tests could also be designed to guide antibiotic therapy for treating gonorrhea.

## Abstract (precis)

Neisseria gonorrhoeae causes the sexually transmitted infection gonorrhea, which is most commonly diagnosed using a DNA-based detection method that does not require growth and isolation of N. gonorrhoeae in the laboratory. This is problematic because the rates of antibiotic resistance in N. gonorrhoeae are increasing, but without isolating the organism in the clinical laboratory, antibiotic susceptibility testing cannot be performed on strains recovered from clinical specimens. We observed an increase in the frequency of urine cultures growing N. gonorrhoeae after we implemented a total laboratory automation system for culture in our clinical laboratory. Here, we report on the rates of resistance to multiple historically used, first-line, and potential future-use antibiotics for 64 N. gonorrhoeae isolates. We found that the rates of antibiotic resistance in our isolates were comparable to national rates. Additionally, resistance to specific antibiotics correlated closely with the presence of genetic resistance genes, suggesting that DNA-based tests could also be designed to guide antibiotic therapy for treating gonorrhea.

## INTRODUCTION

Neisseria gonorrhoeae, the microorganism that causes the sexually transmitted disease gonorrhea, infects approximately one million people in the United States annually (1, 2). Historically, penicillin and doxycycline have been used to treat gonorrhea; however, the emergence of N. gonorrhoeae strains resistant to these antibiotics in the 1980s prompted the implementation of alternative treatment strategies. While the antimicrobial regimen of choice recommended in clinical guidelines has varied over the past decades, today, single-dose ceftriaxone plus azithromycin combination therapy is the standard of care for the treatment of gonorrhea in the United States and much of the world (3–7). With the advent of direct-from-specimen nucleic acid amplification testing (NAAT), gonorrhea can now be diagnosed and treated in a single clinic or emergency room encounter. NAAT is rapid, highly sensitive, and specific; as such, it has become the standard of care for diagnosing gonorrhea and has largely replaced culture-based diagnosis of N. gonorrhoeae, which is slow and suffers from poor sensitivity (8, 9). Importantly however, culture is a prerequisite for performing antimicrobial susceptibility testing (AST) on an organism, and recent trends suggest that N. gonorrhoeae is becoming increasingly resistant to antibiotics, including azithromycin and ceftriaxone (7, 10–14) (for a review, see reference 15).

**FIG 1:**
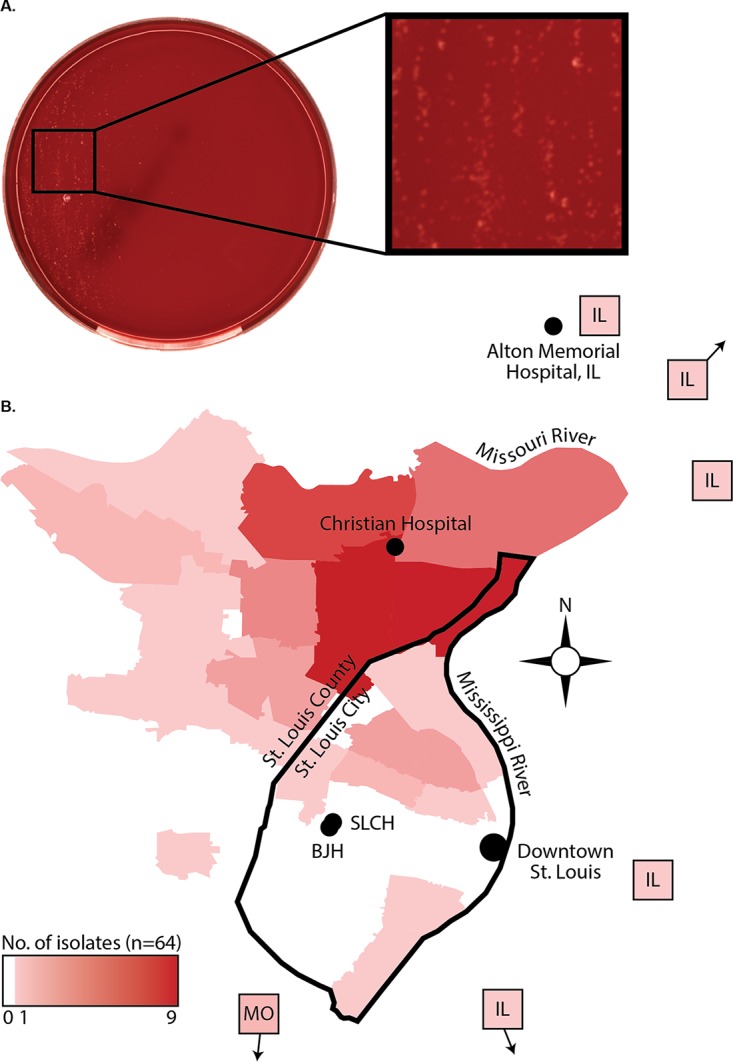
Recovery of N. gonorrhoeae from urine cultures in the St. Louis area. (A) Blood agar plate showing growth of N. gonorrhoeae from routine urine culture performed on a total laboratory automation system (the BD KiestraTLA). Colonies appear nonhemolytic, pin-point, and clear/gray/white in color. (B) Map of the St. Louis metropolitan area showing the primary residence of the patient from which each isolate was obtained at the zip code level or with an arrow indicating the direction of zip codes that are not on the map. Heat mapping highlights the number of patients per zip code, with darker red indicating more patients per zip code. BJH, Barnes-Jewish Hospital; SLCH, St. Louis Children’s Hospital.

Within the past 3 years, the clinical microbiology laboratory at Barnes-Jewish Hospital (affiliated with Washington University in St. Louis)—a central laboratory for 5 hospitals in a 13-hospital health care system—implemented a total laboratory automation system for culture-based microbiology (BD KiestraTLA; Becton-Dickinson). After these changes, the laboratory observed a dramatic increase in the number of routine urine cultures from which fastidious microorganisms were recovered (16). This included a large increase in routine urine cultures growing N. gonorrhoeae, which typically does not grow on blood agar medium under atmospheric oxygen tension commonly used for conventional urine culture (Fig. 1A). Here, we use whole-genome sequencing and AST to characterize the epidemiology and antimicrobial resistance patterns and establish genotype-phenotype correlations for 64 N. gonorrhoeae isolates from patients in the St. Louis area.

## RESULTS

### Sample set and patient demographics.

Of 74 isolates initially identified as N. gonorrhoeae during routine workup by matrix-assisted laser desorption ionization−time of flight mass spectrometry (MALDI-TOF MS) (BioTyper) in the clinical microbiology laboratory at Barnes-Jewish Hospital between November 2015 and October 2017, 64 met all inclusion criteria for this study, including recovery from cryopreservation, confirmed identification as N. gonorrhoeae by MALDI-TOF MS (Vitek MS) after subculture, and complete patient records. Of note, there was 96% agreement between the two MALDI-TOF MS methods.

The majority of the isolates in our sample set came from young black men living in North St. Louis, MO. All were symptomatic with some degree of urogenital discomfort (e.g., “urethral discharge” or “dysuria”); urine cultures were performed as part of a general urogenital tract infection workup at the discretion of the treating physician. The mean age of patients was 27 years old (standard deviation [SD], 9.6 years), but ages ranged from 15 to 58 years of age (Table 1). Of 64 (93.8%) patients, 60 were male, and 59/64 (92.2%) of patients were black. Two black women, two white women, and three white men were also represented in our data set. Of 64 isolates, 54 (84.4%) isolates came from patients residing in zip codes from the predominantly African American community of North St. Louis (Fig. 1B). Isolates from other locations were rare and originated from patients scattered throughout more demographically heterogeneous neighborhoods in South St. Louis (3/64 [4.7%]) or St. Louis suburbs in Illinois (5/64 [7.8%]).

**TABLE 1:** Demographics, diagnosis, and antimicrobial therapy for study subjects (*n = *64)

Characteristic^*a*^	No. of subjects (%) or parameter value
Age (yr)	
Mean	27.0
Range	15 − 58
SD	9.6

Sex	
Male	60 (93.8)
Female	4 (6.3)

Race	
Black	59 (92.2)
White	5 (7.8)

Treatment regimen	
Azithromycin (1 g) + ceftriaxone (250 mg)	34
Azithromycin (1 g) + ceftriaxone (250 mg) + metronidazole (2 g)	23 (35.9)
Azithromycin (1 g) + ceftriaxone (250 mg) + ciprofloxacin (500 mg BID for 7 days)	1 (1.6)
Ciprofloxacin (500 mg BID for 14 days)	1 (1.6)
Cephalexin 500 mg BID for 7 days^*b*^	1 (1.6)
Azithromycin (1 g) + TMP-SMX	1 (1.6)
Metronidazole (2 g) only^*c*^	1 (1.6)
Treatment information not available	2 (1.6)

Neisseria gonorrhoeae NAAT test result	
Positive for N. gonorrhoeae (of tested subjects)	59 (98.3)
Negative for N. gonorrhoeae (of tested subjects)	0 (0.0)
Indeterminate (of tested subjects)	1 (1.7)
NAAT not performed (of total subjects)	4 (6.3)

Concurrent STI	
None documented	37 (57.8)
Chlamydia trachomatis	18 (28.1)
Trichomonas vaginalis	2 (3.1)
HIV	2 (3.1)
HCV	1 (1.6)
Not screened or data not available	4 (6.3)

Specimen source	
Urine sample	58 (90.6)
Genital swab	4 (6.3)
Wound swab	1 (1.6)
Ocular swab	1 (1.6)

Culture method	
Kiestra TLA	52 (81.3)
Conventional microbiology workflow	12 (18.8)

a Abbreviations: BID, twice a day; TMP-SMX, trimethoprim-sulfamethoxazole.

b Complicated by pyelonephritis.

c This patient, treated with metronidazole only, presented 2 weeks later and was treated with azithromycin (1 g) plus ceftriaxone (250 mg).

Over the time frame when this study was conducted, N. gonorrhoeae was isolated from 0.064% of all urine cultures performed, representing 0.3% of all uropathogens recovered (16). NAAT was the predominant method of gonorrhea diagnosis, with 60/64 (93.8%) isolates having a corresponding NAAT test at the time of specimen collection; all were positive except for one that was “indeterminate.” The converse was difficult to assess, given the high prevalence of gonorrhea within our laboratory’s catchment area and the superior sensitivity of NAAT over culture as a primary method for diagnosis. Therefore, we were unable to estimate the percentage of specimens that were positive by NAAT but negative by urine culture. Most patients (37/64 [57.8%]) had no concurrent sexually transmitted infections (STIs). However, concurrent infections (as documented via review of the electronic medical record) with Chlamydia trachomatis (18/64 [28.1%]), Trichomonas vaginalis (2/64 [3.1%]), HIV (2/64 [3.1%]), and hepatitis C virus (HCV) (1/64 [1.6%]) were identified in a subset of patients. At the time of presentation, 57/64 (89%) individuals received standard-of-care antimicrobial therapy for gonorrhea: single-dose azithromycin (1 g) plus ceftriaxone (250 mg) (Table 1).

### Antimicrobial susceptibility testing of N. gonorrhoeae isolates.

Given recent reports of increasing antibiotic resistance among N. gonorrhoeae isolates, we evaluated resistance to several antibiotics in our sample set. We assessed antibiotics from the tetracycline, quinolone, beta-lactam, and macrolide classes that were either used historically to treat gonorrhea or are used currently as first-line agents for treating gonorrhea or have been proposed as future therapies for treating multidrug-resistant N. gonorrhoeae (17).

**FIG 2:**
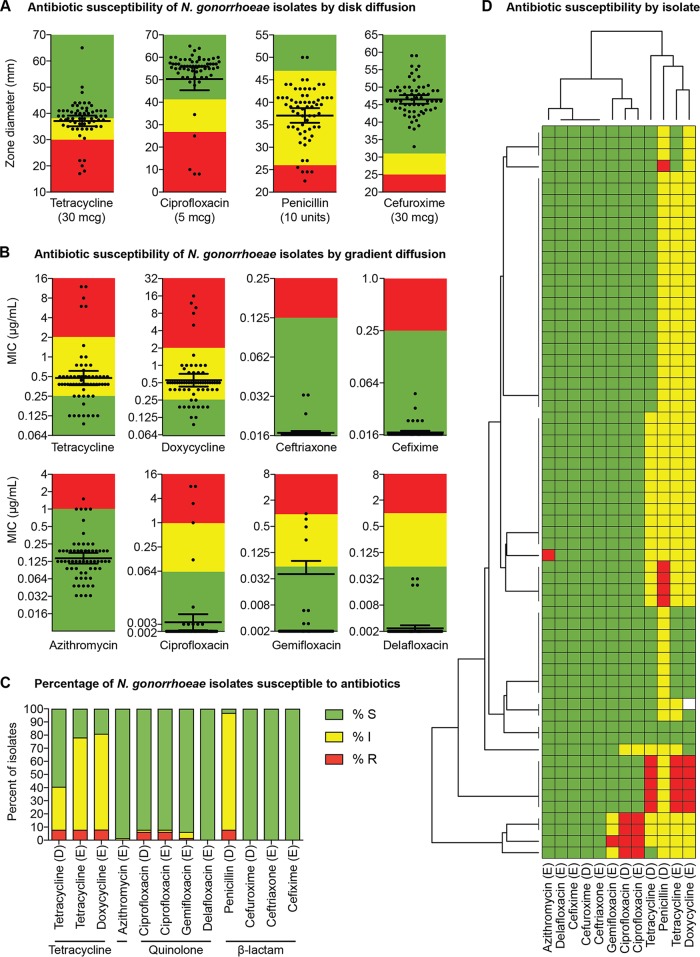
Antimicrobial susceptibility of N. gonorrhoeae isolates. (A and B) Clinical isolates (*n* = 64) were subjected to AST via the disk diffusion method (A) or gradient diffusion (i.e., “Etest”) method (B). Zone-of-inhibition diameters (A) and MICs (B) are shown. Each black circle represents the value for one isolate, and the geometric mean (horizontal black bar) with 95% confidence interval (error bars) for the different treatment groups are shown. For isolates with MICs that were off the measurable scale, a value of 65 mm (disk) or the lowest value (gradient strip) was assigned. Throughout the figure, the breakpoints for each antibiotic are shown by colors: green, susceptible; yellow, intermediate; red, resistant (except for azithromycin, where green represents “wild-type” and red represents “non-wild type.” CLSI breakpoints were used with the following exceptions: doxycycline breakpoints were inferred from tetracycline; gemifloxacin breakpoints were inferred from ciprofloxacin; delafloxacin breakpoints were inferred from ciprofloxacin; and EUCAST breakpoints were used for ceftriaxone. (C) The percentages of isolates that are susceptible (S), intermediate (I), and resistant (R) are shown with the method of susceptibility testing for each drug denoted by a “D” (for disk diffusion) or an “E” (for Etest/gradient diffusion methods) in parentheses after the drug (note that susceptibility results for doxycycline were available for only 63 isolates). (D) Each row in panel D shows the susceptibility profile of a single isolate, with isolates hierarchically clustered by Euclidean distance after assigning a score to each isolate (scores were assigned as follows: 3 for resistant, 2 for intermediate, and 1 for susceptible) to create a matrix. The dendrogram on the left side represents clustering of the individual isolates (*n* = 64), and the dendrogram above the clustermap represents clustering of the antibiotics (*n* = 12). The clustermap was generated in python3 with seaborn (https://seaborn.pydata.org/). The method of susceptibility testing for each drug is denoted by a “D” (for disk diffusion) or an “E” (for Etest/gradient diffusion methods). (Note that susceptibility results for doxycycline were available for only 63 isolates.).

We first assessed the distribution of MICs (or zone diameters) displayed by N. gonorrhoeae isolates against each antibiotic. For penicillin, tetracycline, and doxycycline, isolates formed a multimodal distribution of MICs (or zone diameters) that roughly corresponded to the susceptible (S), intermediate (I), and resistant (R) categories of antimicrobial susceptibility (Fig. 2A and B). A bimodal distribution of MICs was also observed for azithromycin, with a group of isolates straddling the lower bound of the clinical breakpoint. In contrast, the susceptibility of isolates to cephalosporins was distributed about a mean that resided deep within the susceptible category. This was also true for the quinolone class of antibiotics, with the exception of four outliers that displayed increased MICs to ciprofloxacin, gemifloxacin, and delafloxacin.

The greatest percentage of isolates with nonsusceptible phenotypes (i.e., resistant or intermediate) were found for penicillin (96.9%: 7.8% R, 89.0% I), doxycycline (81.0%), and tetracycline (40.6% by disk diffusion; 78.1% by gradient diffusion) (Fig. 2C). Isolates were highly susceptible to first-line therapy: only 1 isolate of 64 isolates (1.6%) was non-wild-type to azithromycin, and none of the 64 isolates (0%) were resistant to ceftriaxone (or any of the other cephalosporins tested). Among quinolone antibiotics, the highest rate of nonsusceptibility was seen for ciprofloxacin (5/64 [7.8%]), with four of the ciprofloxacin-resistant isolates also testing nonsusceptible to gemifloxacin (4/64 [6.25%]) and displaying increased MICs for delafloxacin. These four isolates also tested nonsusceptible to penicillin, doxycycline, and tetracycline, indicative of a multidrug-resistant phenotype (Fig. 2D).

**FIG 3:**
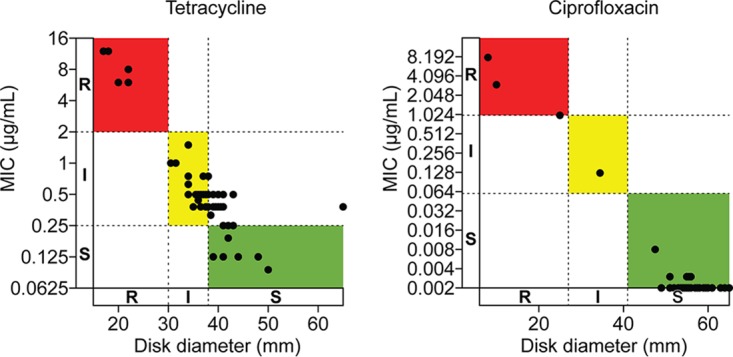
Gradient diffusion versus disk diffusion methods for antimicrobial resistance testing of N. gonorrhoeae isolates. The susceptibility of each isolate to tetracycline and ciprofloxacin was assessed by gradient diffusion and disk diffusion for each antibiotic. MIC, as determined by gradient diffusion, is shown on the *y* axis (natural log scale) with dashed lines denoting the breakpoints between susceptible (S), intermediate (I), and resistant (R). Disk diameter is shown on the *x* axis. Colors demarcate zones of categorical concordance between the two test methods (green for S, yellow for I, red for R).

We also examined categorical agreement between the disk and gradient diffusion methods for tetracycline and ciprofloxacin. Isolates displayed a wide range of susceptibilities to tetracycline, with zone diameter correlating strongly with MIC (*R*^2^ = 99% using a least-squares nonlinear fit model; Fig. 3). However, this corresponded to only 40/64 isolates (62.5%) having categorical agreement, with the remaining 24/64 isolates (37.5%) testing susceptible by disk diffusion but intermediate by gradient diffusion. Nonetheless, there were zero major or very major errors for tetracycline disk versus gradient diffusion methods. Isolates with resistance to ciprofloxacin were uncommon; nonetheless, the correlation between zone diameter and MIC was very high (*R*^2^ = 99%), as was categorical agreement (100%).

### Genetic determinants of antimicrobial resistance in N. gonorrhoeae isolates.

**FIG 4:**
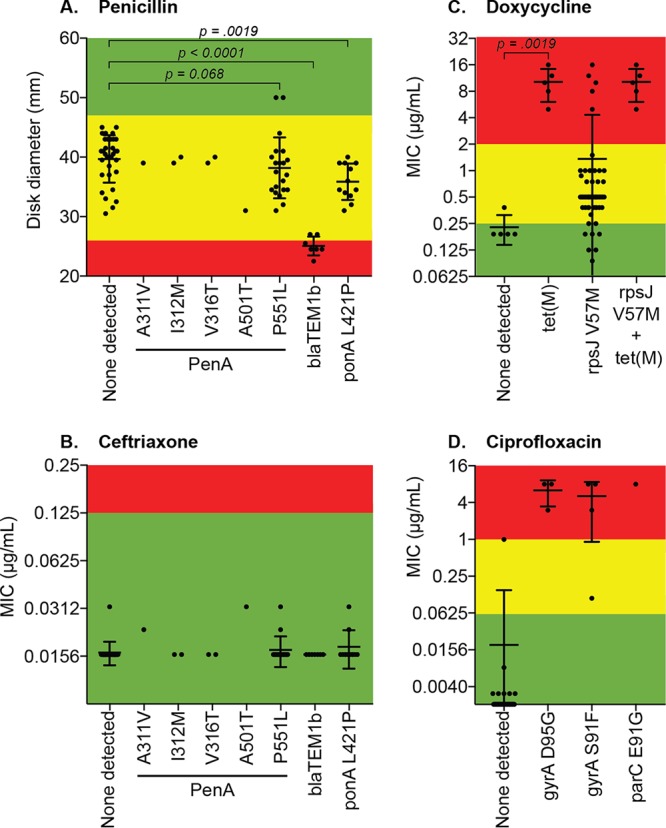
Correlation of resistance phenotypes with known resistance determinants in N. gonorrhoeae isolates. Whole-genome sequencing was performed on all clinical isolates (*n* = 64), and common antimicrobial resistance determinants were identified. Each graph shows the zone of inhibition or MIC for a drug with the corresponding resistance determinants displayed on the *x* axis. Only determinants that were present in one or more isolates are shown; if an isolate harbored more than one resistance determinant, it is displayed for each determinant. Colors denote breakpoints for each antibiotic: green, susceptible; yellow, intermediate; red, resistant. *P* values show the results of Mann-Whitney U tests between selected groups. (A) Penicillin; (B) ceftriaxone (results for cefuroxime and cefixime were similar); (C) doxycycline (results for tetracycline were virtually identical); (D) ciprofloxacin (results for delafloxacin and gemifloxacin were similar).

For most of the antibiotics we tested, the zone sizes (or MICs) were not normally distributed (as measured by the D’Agostino-Pearson omnibus normality test; Fig. 2A and B)—a potential indication of acquired resistance genes and/or mutations in a subset of isolates. To explore this observation further, we performed whole-genome sequencing on each isolate. The ResFinder and PointFinder databases were used to identify known genes and mutations (collectively referred to as “resistance determinants”), respectively. We detected several penicillin resistance determinants; however, only acquisition of the *bla*_TEM-1b_ beta-lactamase gene was consistently associated with penicillin resistance and was found in 100% of penicillin-resistant isolates (Fig. 4A). As expected, the presence of *bla*_TEM-1b_ was not associated with increased cephalosporin MICs (or reduced zone sizes), nor were any of the other resistance determinants we examined (Fig. 4B).

Within the tetracycline class, acquisition of *tet*(M) was highly associated with resistance to both tetracycline and doxycycline, with all five (100%) resistant isolates containing *tet*(M) (Fig. 4C). All five isolates resistant to tetracycline also had the *rpsJ* V57M mutation, but isolates containing the *rpsJ* mutation yet lacking *tet*(M) were not resistant.

Only one of five isolates with increased MICs to azithromycin contained an established multidrug resistance determinant (an *mtrR* promoter mutation), although this particular isolate still met the definition of “wild type.”

The genotype-phenotype correlation for the quinolone antibiotics was less straightforward. All four isolates with known resistance mutations in *gyrA* and *parC* were resistant to ciprofloxacin, with the exception of one isolate with a *gyrA* S91F mutation which was categorized as “intermediate” to ciprofloxacin (MIC = 0.125 μg/ml). This particular isolate contained only the *gyrA* S91F mutation whereas the other three isolates contained a *gyrA* D95G mutation in addition to the *gyrA* S91F mutation (Fig. 4D). Accordingly, the isolate with the lone *gyrA* S91F mutation was also susceptible to gemifloxacin and delafloxacin, whereas the other isolates containing multiple quinolone resistance determinants tested “intermediate” to gemifloxacin and had increased MICs to delafloxacin. Finally, one isolate contained no known quinolone resistance determinants but repeatedly tested as resistant to ciprofloxacin with increased MICs to gemifloxacin and delafloxacin.

### Phylogenetic analysis of N. gonorrhoeae isolates.

**FIG 5:**
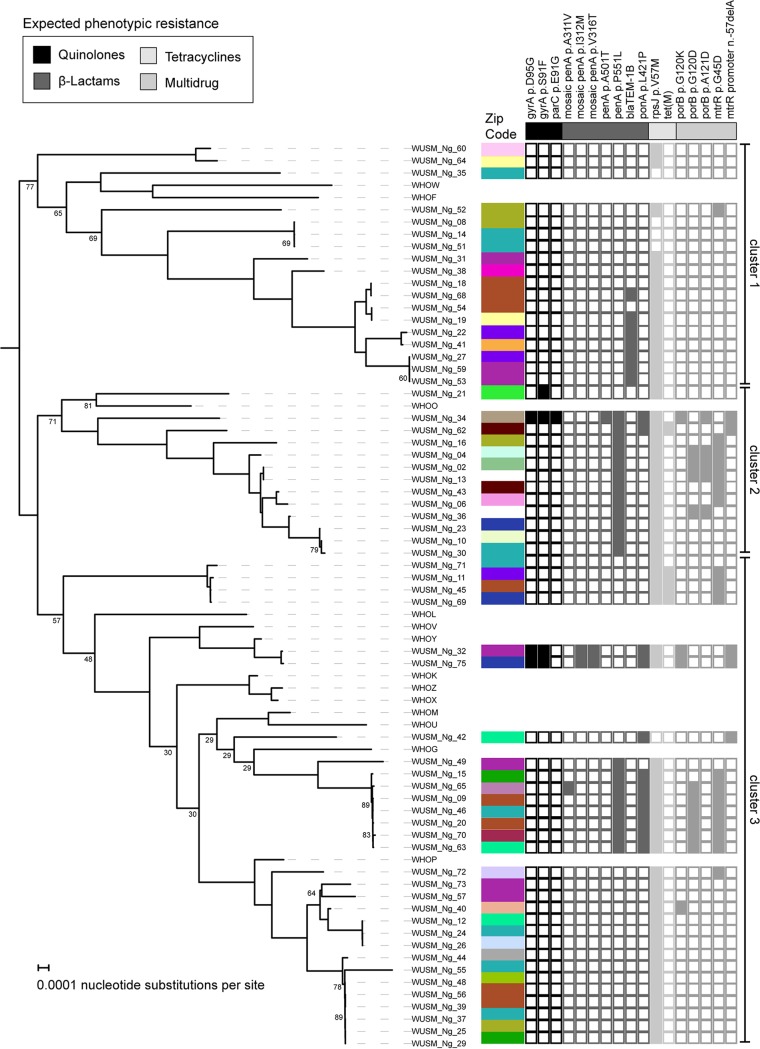
Core genome phylogenetic analysis with geographic information and antibiotic resistance determinants. A maximum likelihood phylogenetic tree was constructed based upon the 1,563 core gene alignment created in PRANK. Bootstrap support values below 90% are shown at the corresponding branch-points. The geographic origin of each isolate (zip code) is denoted by color adjacent to the isolate name. The presence or absence of an antibiotic resistance determinant in a particular isolate is denoted by a colored or blank square, respectively. If resistance determinants in the ResFinder or PointFinder databases were not found in any of the isolates, they are not shown in the figure.

To examine relationships between isolates, we constructed a core genome phylogeny using PRANK and raxML on the 1,563 genes shared by the genomes collected in our investigation and the WHO reference strains (18, 19) (Fig. 5). Midpoint rooting of the phylogenetic tree showed that the collected isolates fell within three major clades. WHOF/WHOW strains were in the first cluster, WHOO strain was in the second cluster, and the remaining WHO strains were in the third cluster. To identify putative transmission clusters, we labeled each isolate with its corresponding zip code; however, transmission clusters were not identified. However, we observed associations between resistance determinants and position of the isolates on the phylogenetic tree. *bla*_TEM-1b_ was located only in isolates within the first cluster, and *tet*(M) was found only in the second cluster.

## DISCUSSION

Because NAAT is the standard of care for diagnosis of N. gonorrhoeae, clinical isolates for detailed phenotypic and genotypic characterization are scarce. New culture methods facilitated enhanced recovery of N. gonorrhoeae isolates in our laboratory, and subsequently, we characterized 64 patient isolates, including analysis of demography, geography, AST, and whole-genome sequencing.

As expected, the rates of antibiotic resistance to historically used antibiotics such as penicillin and doxycycline were high and were correlated with the presence of the *bla*_TEM-1b_ and *tet*(M) genes, respectively. However, almost no resistance to current first-line antibiotics, including azithromycin or drugs in the cephalosporin class was detected, consistent with low rates of resistance to first-line antibiotics reported throughout the United States (1). We also found a relatively low rate of ciprofloxacin resistance (6%) in the N. gonorrhoeae isolates in our study, which is in contrast to the rapid increase in resistance to quinolone antibiotics seen nationwide (approximately 20 to 40% of isolates in 2016, depending on location) (1, 20). However, several of the ciprofloxacin-resistant isolates we identified contained a combination of mutations in the *gyrA* and *parC* genes known to confer high-level resistance to ciprofloxacin (15, 21). These isolates also displayed increased MICs to quinolones that have been proposed as next-generation agents to combat multidrug-resistant N. gonorrhoeae—gemifloxacin and delafloxacin—suggesting that determinants of resistance to ciprofloxacin may be poised to rapidly acquire resistance and/or exhibit cross-resistance to other antibiotics in the quinolone class (17, 21). Furthermore, all isolates containing quinolone resistance determinants were also resistant to antibiotics from at least two other classes, suggesting that quinolone resistance may be a marker of multidrug resistance and that this association could be a useful tool as a surrogate for multidrug resistance to incorporate into future molecular diagnostic methods. One limitation of the antimicrobial resistance characterization in our study is that opportunistically available isolates from routine urine cultures could have biased our recovery of antibiotic-resistant organisms.

Given the AST profiles of the N. gonorrhoeae isolates in our study and considering that the majority of patients (57/64 [89%]) received single-dose azithromycin (1 g) plus ceftriaxone (250 mg) at the time of presentation (Table 1), we predict that the cure rate for gonorrhea using empirical treatment in our patient population is high. The high percentage of N. gonorrhoeae-positive cultures that were concurrently diagnosed by NAAT (Table 1) also implies that few additional cases are diagnosed by urine culture. Taken together, these findings suggest that the current standard of care for N. gonorrhoeae diagnosis, NAAT, continues to be appropriate in our patient population, except in cases of suspected treatment failure when cultured isolates are necessary for AST.

Nevertheless, global trends in N. gonorrhoeae antimicrobial resistance portend the need for AST methods that are more accessible than the agar dilution method, which is labor-intensive and performed only in reference laboratories. Studies comparing agar dilution to gradient diffusion devices for several antibiotics have demonstrated acceptable performance characteristics of gradient diffusion devices compared to agar dilution for several antibiotics (22–24). We found a high degree of correlation between gradient diffusion and disk diffusion AST results for tetracycline and ciprofloxacin, as reflected by the observed *R*^2^ values, suggesting that disk diffusion may also be an acceptable and accessible method of tetracycline and ciprofloxacin resistance testing in N. gonorrhoeae*;* however, this may require future reassessment of the tetracycline breakpoints. Although no major or very major errors occurred, 24/64 isolates (37.5%) tested susceptible by disk diffusion but intermediate by gradient diffusion.

Given the ongoing challenges of performing AST on N. gonorrhoeae isolates in the clinical setting, direct-from-specimen genotyping, especially when multiplexed with NAAT, could have a major impact on guiding the choice of antimicrobial therapy (25). Indeed, a recent proof-of-concept study showed that ciprofloxacin therapy was 100% effective in curing gonorrhea caused by strains lacking resistance-conferring *gyrA* mutations (26). Our study adds to a growing body of literature demonstrating strong associations between specific genotypic markers and resistance to several antibiotics [i.e.*, bla*_TEM-1b_ for penicillin, *tet*(M) for tetracyclines, and *gyrA* with or without *parC* for quinolones], suggesting that additional genetic targets—via a targeted or whole-genome sequencing approach—may enable the effective use of antimicrobials that are no longer appropriate for use as empirical therapy (27–29). However, our identification of one ciprofloxacin-resistant isolate lacking any known quinolone resistance determinants highlights the current limitations of this approach and the need for ongoing efforts to interrogate the resistome of N. gonorrhoeae strains and expand the repository of genetic determinants of phenotypic resistance.

## MATERIALS AND METHODS

### Ethics statement.

Prior to study initiation, this investigation was reviewed by the institutional review board at the Washington University School of Medicine and approved with a waiver of consent.

### Clinical isolates.

Growth of bacterial isolates from clinical specimens identified as N. gonorrhoeae by matrix-assisted laser desorption ionization−time of flight mass spectrometry (MALDI-TOF MS) using the BioTyper (Bruker Corporation) were frozen at −80°C in reconstituted powdered milk. Prior to antimicrobial susceptibility testing (AST) and sequencing, isolates were thawed and subcultured twice on chocolate agar (Hardy Diagnostics) incubated at 35°C ± 2°C with 5% CO_2_, and the organism identification was confirmed with Vitek MS MALDI-TOF MS (bioMérieux). Patient characteristics such as age, sex, coinfections, and zip code of residence, were obtained from retrospective review of the electronic medical record.

### Antimicrobial susceptibility testing.

After two subcultures, plates containing GC II agar with IsoVitaleX enrichment (Becton-Dickinson) were inoculated with a 0.5 McFarland direct-colony suspension of each isolate. The plates were incubated at 35°C ± 2°C with 5% CO_2_ and read by two independent readers after 24 h of incubation. Quality control was performed each day of testing (N. gonorrhoeae ATCC 49226 and Escherichia coli ATCC 25922).

For AST results, results from two independent readers were averaged and then rounded up to the nearest doubling dilution (gradient diffusion method only). For isolates with AST values that were off the measurable scale, a value of 65 mm (disk) or the lowest value on the gradient strip was assigned as the value for the purpose of analysis. Categorical results were determined by comparing these results to established clinical breakpoint criteria from the Clinical and Laboratory Standards Institute (CLSI) M100-S29 document (30) with the following exceptions. Doxycycline breakpoints were inferred from tetracycline (30). Gemifloxacin and delafloxacin breakpoints were inferred from ciprofloxacin (30). The recently published CLSI M100-S29 breakpoint was used for azithromycin (30). The European Union Committee on Antimicrobial Susceptibility Testing (EUCAST) breakpoints (31) were used for ceftriaxone. Additional information on AST devices, including manufacturer and breakpoint criteria, can be found in Table S1 in the supplemental material.

10.1128/mSphere.00373-19.1 TABLE S1 Information on antimicrobial susceptibility testing performed in this study. Download Table S1, XLSX file, 0.01 MB. Copyright © 2019 Bailey et al. 2019 Bailey et al. This content is distributed under the terms of the Creative Commons Attribution 4.0 International license.

### Whole-genome sequencing and analysis.

Sample preparation, sequencing, and sequence analysis were performed as described previously (32) using 12 World Health Organization (WHO) reference genomes (18) (Table S2). Genomic DNA was obtained from pure N. gonorrhoeae cultures using the QIAmp BiOstic Bacteremia DNA kit (Qiagen, Germantown, MD, USA), and 0.5 ng of genomic DNA was used to create sequencing libraries with the Nextera kit (Illumina, San Diego, CA, USA) (33). Samples were pooled and sequenced on an Illumina NextSeq to obtain 2 × 150 bp reads. The raw reads and Illumina adapters were removed with trimmomatic v.38, and contaminating DNA was removed with DeconSeq v.4.3 (34, 35). Processed reads were used to *de novo* assemble scaffolds with SPAdes v3.13.0 (36). Quality statistics were obtained with QUAST v4.5, and protein-coding sequences were identified with prokka v1.12 (Table S3) (37, 38). Of 64 genomes, 61 passed *in silico* quality assessment and were used in genomic analysis. We additionally gathered World Health Organization (WHO) reference N. gonorrhoeae genomes and identified all protein-coding sequences with prokka (18). Coding sequences were clustered at 95% identity with roary v3.12.0, and the 1,563 core genes were used to create a core genome alignment with PRANK v1.0 (19, 39). The core genome alignment was converted to a maximum likelihood phylogenetic tree with RAxML and viewed in iToL with an overlay showing zip code and identified resistance determinant (40, 41). Vertically transmitted mutations associated with antimicrobial resistance were identified with the N. gonorrhoeae database using PointFinder v4.0 on the scaffolds.fasta file from SPAdes. Acquired antibiotic resistance genes were identified with ResFinder v4.0 (42).

10.1128/mSphere.00373-19.2 TABLE S2 Genomes analyzed in this study. Download Table S2, XLSX file, 0.07 MB. Copyright © 2019 Bailey et al. 2019 Bailey et al. This content is distributed under the terms of the Creative Commons Attribution 4.0 International license.

10.1128/mSphere.00373-19.3 TABLE S3 Quality metrics for genomic assemblies in this study. Download Table S3, XLSX file, 0.04 MB. Copyright © 2019 Bailey et al. 2019 Bailey et al. This content is distributed under the terms of the Creative Commons Attribution 4.0 International license.

### Data analysis.

Data were tabulated in Excel (Microsoft), where demographic and summary statistics were calculated. Data were then imported into Prism version 7 (GraphPad) for graphing and group comparisons. All statistical methods are described in the corresponding figure legends. Color overlays and final figures were prepared in Illustrator CC 2017 (Adobe).

### Data availability.

All genomes used in this study have been deposited into the NCBI Whole Genome Shotgun Database associated with BioProject accession no. PRJNA504667.
